# Prognostic indicators in the World Health Organization’s algorithm for seriously ill HIV-infected inpatients with suspected tuberculosis

**DOI:** 10.1186/s12981-018-0192-0

**Published:** 2018-02-12

**Authors:** Rulan Griesel, Annemie Stewart, Helen van der Plas, Welile Sikhondze, Marc Mendelson, Gary Maartens

**Affiliations:** 1 Division of Clinical Pharmacology, Department of Medicine, UCT Faculty of Health Sciences, Anzio Road, Observatory, Cape Town, 7925 South Africa; 2 Division of Infectious Diseases and HIV Medicine, Department of Medicine, University of Cape Town, Cape Town, South Africa

**Keywords:** HIV, Tuberculosis, *Pneumocystis jirovecii* pneumonia, Bacterial pneumonia, Prognosis, Mortality, WHO algorithm

## Abstract

**Background:**

Criteria for the 2007 WHO algorithm for diagnosing tuberculosis among HIV-infected seriously ill patients are the presence of one or more danger signs (respiratory rate > 30/min, heart rate > 120/min, temperature > 39 °C, and being unable to walk unaided) and cough ≥ 14 days. Determining predictors of poor outcomes among HIV-infected inpatients presenting with WHO danger signs could result in improved treatment and diagnostic algorithms.

**Methods:**

We conducted a prospective cohort study of inpatients presenting with any duration of cough and WHO danger signs to two regional hospitals in Cape Town, South Africa. The primary outcome was all-cause mortality up to 56 days post-discharge, and the secondary outcome a composite of any one of: hospital admission for > 7 days, died in hospital, transfer to a tertiary level or tuberculosis hospital. We first assessed the WHO danger signs as predictors of poor outcomes, then assessed the added value of other variables selected a priori for their ability to predict mortality in common respiratory opportunistic infections (CD4 count, body mass index (BMI), being on antiretroviral therapy (ART), hypotension, and confusion) by comparing the receiver operating characteristic (ROC) area under the curve (AUC) of the two multivariate models.

**Results:**

484 participants were enrolled, median age 36, 66% women, 53% had tuberculosis confirmed on culture. The 56-day mortality was 13.2%. Inability to walk unaided, low BMI, low CD4 count, and being on ART were independently associated with poor outcomes. The multivariate model of the WHO danger signs showed a ROC AUC of 0.649 (95% CI 0.582–0.717) for predicting 56-day mortality, which improved to ROC AUC of 0.740 (95% CI 0.681–0.800; p = 0.004 for comparison between the two ROC AUCs) with the multivariate model including the a priori selected variables. Findings were similar in sub-analyses of participants with culture-positive tuberculosis and with cough duration ≥ 14 days.

**Conclusion:**

The study design prevented a rigorous evaluation of the prognostic value of the WHO danger signs. Our prognostic model could result in improved algorithms, but needs to be validated.

**Electronic supplementary material:**

The online version of this article (10.1186/s12981-018-0192-0) contains supplementary material, which is available to authorized users.

## Background

Undiagnosed tuberculosis is a major cause of death among hospitalised HIV-infected patients in Africa [1, 2]. The World Health Organization (WHO) recommendations to improve the diagnosis of tuberculosis and facilitate rapid initiation of empiric anti-tuberculosis treatment among adults in high HIV prevalence settings include an algorithm for seriously ill patients presenting with cough and one or more of the following danger signs: respiratory rate > 30/min, heart rate > 120/min, temperature > 39 °C, and being unable to walk unaided [3]. Developing a strong evidence base for predictors of poor outcomes in seriously ill inpatients could result in improved treatment and diagnostic algorithms. Empiric antituberculosis treatment improved 8-week survival of inpatients with WHO danger signs and negative sputum smears, but had no effect on survival in inpatients without danger signs in a Ugandan study; [4] which indicates that the danger signs have prognostic value.

We were unable to find any studies that evaluated predictors of poor outcomes in the WHO algorithm for seriously ill patients. Many studies have evaluated predictors of mortality of the three commonest respiratory opportunistic infections in HIV-infected patients: *Pneumocystis jirovecii* pneumonia (PJP), bacterial pneumonia, and tuberculosis. Most reports of prognostic indicators for PJP include laboratory tests, pulmonary function tests, and/or sophisticated imaging that are not widely available in resource-limited settings. Simple predictors of mortality in PJP include wasting, hypoxia, hypotension, and lymphopenia [5, 6]. The pneumonia severity index performs well at predicting mortality among hospitalized HIV-infected patients with bacterial pneumonia, but it has multiple clinical, laboratory and radiological features [7–9]. CRB-65 is a simple clinical pneumonia severity score, which predicted mortality and hospital stay in patients irrespective of HIV status in one study, [9] but predicted mortality poorly in a Malawian study, which found that male sex, wasting, non-ambulatory functional status, extremes of temperature, and hypotension predicted mortality [10]. Most of the studies that assessed predictors of mortality in patients with HIV-associated tuberculosis were conducted before the availability of antiretroviral therapy (ART) and focused on 6 month mortality, and thus are not informative about predicting short-term mortality in inpatients with newly diagnosed tuberculosis in the ART era. A Ugandan study of hospitalized HIV-infected patients presenting with suspected pneumonia of 2 weeks to 6 months duration, most of whom had tuberculosis, found heart rate > 120/min, respiratory rate > 30/min, oxygen saturation < 90%, and CD4 cell count < 50 cells/µL predicted mortality [11].

We assessed the WHO danger signs together with other prognostic variables selected for their ability to predict mortality in HIV-associated respiratory illness (confusion, hypotension, CD4 count, ART status) as predictors of mortality and poor outcomes in participants fulfilling entry criteria for the WHO algorithm for seriously ill HIV-infected patients with a cough enrolled into a prospective cohort study in Cape Town, South Africa. We enrolled participants with cough of any duration, rather than the WHO recommendation of 2 weeks or more, because studies in high burden countries have shown that acute presentations of pulmonary tuberculosis occur commonly in HIV-infected patients [12, 13]. We have previously reported the development of clinical prediction rules and the use of Xpert MTB/RIF assay to improve the diagnosis of tuberculosis in this cohort [14].

## Methods

### Study population

We conducted a prospective cohort study. The study sites were two regional hospitals serving communities with a high burden of HIV and tuberculosis in Cape Town, South Africa: GF Jooste Hospital from November 2011 to February 2013, when the hospital closed; and Khayelitsha District Hospital from March 2013 until October 2014. Inclusion criteria were: known or newly diagnosed HIV infection, within 24 h of hospital admission, ≥ 18 years of age, cough of any duration, and at least one of the WHO danger signs. Exclusion criteria were: being on anti-tuberculosis therapy, having completed anti-tuberculosis therapy in the previous month, defaulting anti-tuberculosis therapy within the past 6 months, exacerbations of congestive cardiac failure or chronic obstructive pulmonary disease, and inability to produce a spontaneous or induced sputum sample. After discharge from hospital, participants were followed up at 28 and 56 days. Follow up was either a physical visit to hospital or a telephonic consult to assess their clinical response and outcome. During the study period the CD4 criterion for ART initiation was < 350 cells/uL.

### Data collection

Participants enrolled into the study received a standardized work-up to assess the aetiology of their respiratory illness. A standard case record form was used for capturing demographic data, medical history, symptoms, and signs on clinical examination. All participants had a chest radiograph on admission. CD4 counts were done on admission unless a result was available within 6 months prior to admission. Sputum samples were taken from all participants: 1 sample for Gram stain, culture and sensitivity; and 2 samples for smear examination with auramine staining for acid-fast bacilli (AFB) and mycobacterial culture. On 1 sample sent for mycobacterial culture the Cepheid Xpert MTB/RIF assay was performed. If participants were unable to produce sputum spontaneously, sputum induction was performed using an ultrasonic nebulizer and hypertonic saline. Participants had blood taken for a mycobacterial blood culture (BacT/Alert^®^ specimen bottle).

### Study definitions

The case definition of tuberculosis was a positive tuberculosis culture from any site. Anti-tuberculosis therapy was commenced in participants who met microbiological criteria (any one of: positive auramine stain for AFB, positive Cepheid Xpert MTB/RIF assay, positive culture for tuberculosis); and/or radiological criteria (a chest radiograph showing either mediastinal and/or hilar lymph nodes or miliary infiltrates; an abdominal ultrasound showing multiple enlarged lymph nodes and/or multiple splenic hypoechoic lesions and/or a pericardial effusion); and/or or pleural effusions/ascites showing a lymphocytic exudate. Anti-tuberculosis therapy was also commenced if participants showed no clinical improvement after 3–5 days of antibiotic therapy.

The diagnosis of bacterial pneumonia was made clinically with consistent symptoms and evidence of pulmonary consolidation on a chest x-ray. Participants clinically diagnosed with bacterial pneumonia were started on empirical antibiotic therapy with a ß-lactam antibiotic (usually intravenous ceftriaxone). A macrolide was added if participants fulfilled the criteria for severe pneumonia (CRB-65 score > 2) [15].

PJP was diagnosed if the following were present: cough duration ≤ 12 weeks, bilateral interstitial infiltrates on chest radiograph, and hypoxia at rest or on exertion (> 5% drop in oxygen saturation). Weight based high dose trimethoprim-sulfamethoxazole and corticosteroid therapy were initiated in these participants.

### Statistical analysis

The primary outcome was all-cause mortality from admission to 56 days post-discharge. The secondary outcome was a composite of any one of: hospital admission for > 7 days, died in hospital, transfer to a tertiary level hospital, or transfer to a tuberculosis hospital. In Cape Town patients with tuberculosis are only admitted to tuberculosis hospitals if they are too ill for ambulatory care (unless they have extensively drug-resistant tuberculosis), therefore admission to a tuberculosis hospital reflects need for prolonged hospitalisation.

Analyses were performed using STATA 12.1 (StataCorp, Texas, USA). Missing data were imputed using chained equations and five iterations. We checked the results of the imputation by comparing a complete case analysis with the imputation set.

Multivariate logistic regression analysis was performed including variables in the multivariate models selected a priori for their ability to predict mortality in common respiratory opportunistic infections and their implementability in resource-limited settings. We first investigated the primary outcome of all-cause mortality within the 56 days of follow-up post-discharge. We tested two multivariate models for this outcome: the WHO multivariate model (variables were the WHO danger signs); and the augmented multivariate model (variables were the WHO danger signs, and the following: CD4 count, body mass index (BMI), being on ART, hypotension (systolic blood pressure < 90 mmHg or diastolic blood pressure ≤ 60 mmHg), and confusion). We compared the area under the curve (AUC) for the receiver-operating characteristic (ROC) of the two models. We then investigated the composite secondary outcome in a similar manner. A sub-analysis on all participants with culture positive tuberculosis was performed using the same approach for each of the specified outcomes.

## Results

### Participant characteristics

**Fig. 1:**
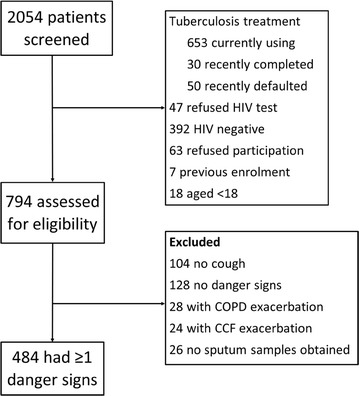
Flow diagram for participant inclusion into the study

Two thousand and fifty-four patients were screened for inclusion into the study; 484of whom met the inclusion criteria and were enrolled (Fig. 1). The baseline characteristics of the participants are listed in Table 1. The majority of participants had > 1 WHO danger sign at presentation; 23% (111/484) had one, 45% (216/484) had two, 27% (133/484) had three, and 5% (24/484) had all four danger signs. The diagnoses and outcomes of participants are summarised in Table 2. Sixty-four percent (309/484) of participants were started on anti-tuberculosis therapy, of whom 255 (83%) were confirmed on culture. Simultaneous tuberculosis and bacterial pneumonia was diagnosed in 26% (125/484) of participants and simultaneous tuberculosis and PJP was diagnosed in 4% (21/484) of participants.

**Table 1:** Baseline characteristics of 484 participants with cough and one or more of the WHO danger signs

Baseline variables		
Age	Median (IQR)	36 (30 to 42)
Sex (female)	n (%)	317 (66)
BMI (kg/m^2^)	Median (IQR)^a^	20 (18 to 39)
CD4 (cells/µL)	Median (IQR)^b^	89 (34 to 210)
Cough duration	Median days (IQR)^c^	14 (7 to 21)
Cough duration < 14 days	n (%)	186 (39)
Hypotensive^d^	n (%)	119 (25)
Using ART	n (%)	171 (35)
Duration on ART (years)	Median (IQR)	2.3 (0.2 to 5)
Confused	n (%)	88 (18)
WHO danger signs		
Respiratory rate > 30	n (%)	315 (65)
Heart rate > 120	n (%)	383 (79)
Temp > 39 °C	n (%)	81 (17)
Unable to walk unaided	n (%)	259 (54)

^a^17 missing values

^b^1 missing values

^c^2 missing values

^d^Systolic blood pressure < 90 mmHg or diastolic blood pressure ≤ 60 mmHg

**Table 2:** Diagnoses and hospitalization outcomes of 484 participants with cough and the WHO danger signs

Diagnoses^a^		
Tuberculosis	n (%)	255 (53)
Community acquired pneumonia	n (%)	245 (51)
* Pneumocystis jirovecii* pneumonia	n (%)	51 (11)
Other	n (%)	41 (8)
Hospitalization and discharge		
Days in hospital	Median (IQR)	5 (3 to 7)
Discharged alive	n (%)	386 (80)
Transfer to tertiary hospital	n (%)	22 (5)
Transfer to tuberculosis hospital	n (%)	53 (11)
Lost to follow-up	n (%)	37 (8)
Mortality		
Died in hospital	n (%)	23 (5)
Died during follow up	n (%)	36 (8)

^a^Total number of diagnoses > 100% due to coinfections

### Primary outcome

**Fig. 2:**
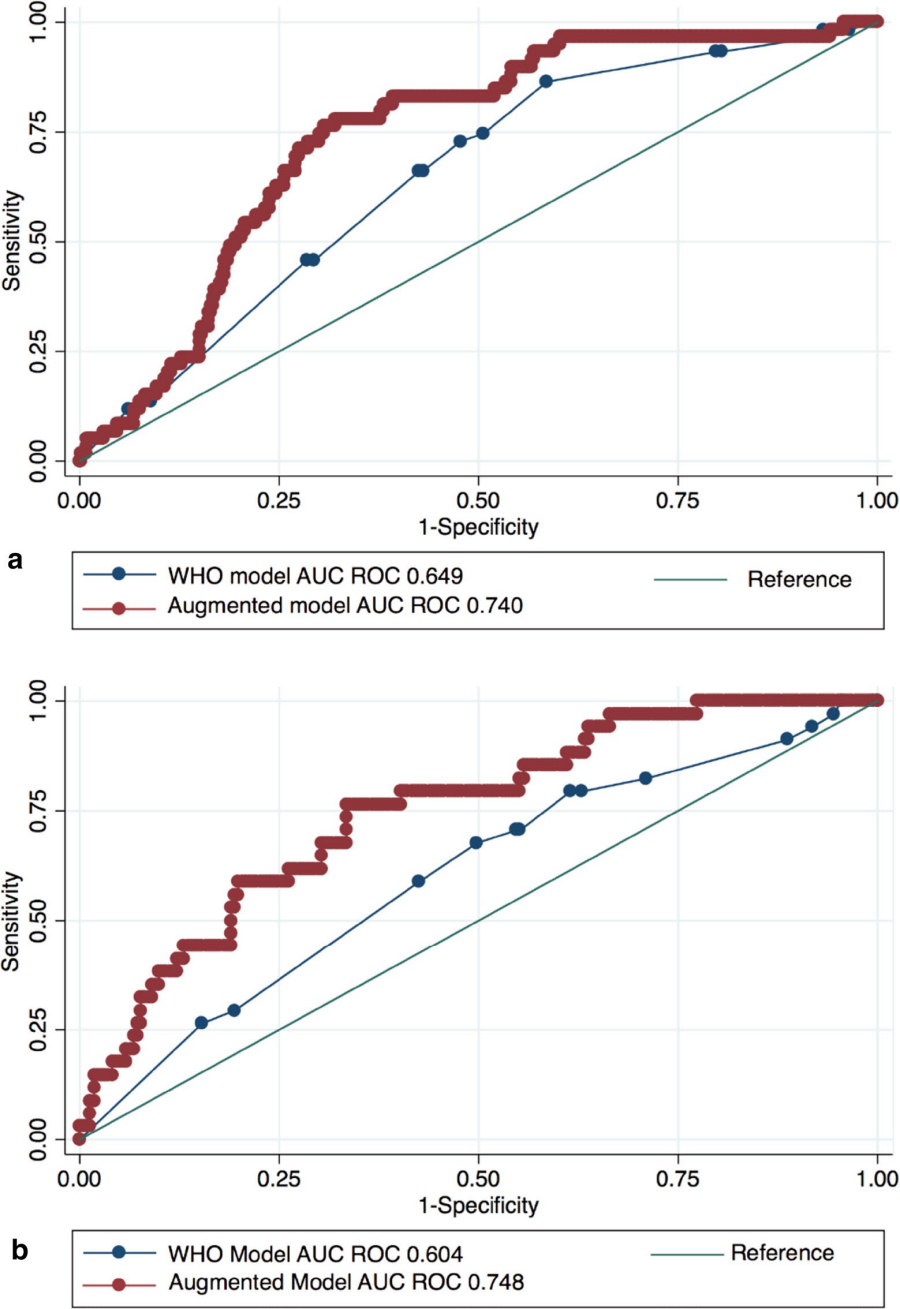
Comparison of area under the curve (AUC) for the receiver operating characteristic (ROC) for the WHO multivariate model (variables were WHO danger signs) and the augmented multivariate model (variables were the WHO danger signs, and the following: CD4 count, body mass index, being on ART, hypotension, and confusion) for predicting death at 56 days post-discharge in all participants (**a**) and in participants with culture-positive tuberculosis (**b**)

The WHO multivariate model showed that being unable to walk unaided was the only WHO danger sign that was independently associated with death at 56 days post-discharge (Table 3). A sub-analysis limited to participants with cough duration of ≥ 14 days (which is the WHO algorithm inclusion criterion) showed similar findings. The augmented multivariate model showed that the following variables were independently associated with death at 56 days post-discharge: a lower CD4 count, being on ART, and being unable to walk unaided (Table 3). Twenty-seven of 59 (46%) participants who died within 56 days post-discharge were on ART; 8 had been on ART for less than 3 months (5 of whom had a CD4 count < 50 cells/µL), and 9 were failing ART with a viral load (VL) of > 1000 RNA copies/mL. The WHO multivariate model showed a ROC AUC of 0.649 (95% CI 0.582–0.717) for predicting death at 56 days post-discharge. The augmented multivariate model showed an improved ROC AUC of 0.740 (95% CI 0.681–0.800; p = 0.004 for comparison between the two ROC AUCs) (Fig. 2a).

**Table 3:** WHO multivariate model (variables were WHO danger signs) and augmented multivariate model (variables were the WHO danger signs, and the following: CD4 count, body mass index, being on ART, hypotension, and confusion) for predicting death at 56 days post-discharge. Imputation set used due to missing CD4 and BMI data

Variable	WHO multivariate model			Augmented multivariate model		
Category	aOR^a^	95% confidence interval	Wald’s *p* value	aOR	95% confidence interval	Wald’s p value
Respiratory rate > 30						
No	Referent group			Referent group		
Yes	1.25	0.68 to 2.29	0.469	1.05	0.55–2.02	0.879
Heart rate > 120						
No	Referent group			Referent group		
Yes	0.68	0.36 to 1.29	0.240	0.59	0.29–1.19	0.138
Temperature > 39 °C						
No	Referent group			Referent group		
Yes	0.66	0.28 to 1.52	0.329	0.67	0.28–1.59	0.364
Unable to walk						
No	Referent group			Referent group		
Yes	2.94	1.58 to 5.47	0.001	2.06	1.06–3.99	0.032
CD4 cells/µL^b^				0.62	0.45–0.86	0.004
BMI (kg/m^2^)^c^				1.02	0.96–1.08	0.535
On ART at admission						
No				Referent group		
Yes				2.54	1.37–4.72	0.003
Hypotensive^d^						
No				Referent group		
Yes				1.10	0.55–2.23	0.782
Confused						
No				Referent group		
Yes				1.48	0.71–3.06	0.295

^a^Adjusted odds ratio

^b^Increase in increments of 100 cells/µL

^c^Increase per 1 kg/m^2^

^d^Systolic blood pressure < 90 mmHg or diastolic blood pressure ≤ 60 mmHg

A sub-analysis of the primary outcome among *Mycobacterium tuberculosis* culture-positive patients, showed that none of the WHO danger signs independently predicted death at 56 days post-discharge (Additional file 1: Table S1). In the augmented multivariate model, death at 56 days post-discharge was independently associated with a lower CD4 count and being on ART (Additional file 1: Table S2). Thirty-four of the 59 participants who died were culture-positive for *M. tuberculosis*, 14 of whom where on ART: 7 were on ART for less than 3 months (5 of whom had a CD4 count < 50 cells/µL), and 4 were failing ART with a viral load (VL) of > 1000 RNA copies/mL. In this sub-analysis of culture confirmed tuberculosis the WHO model showed a ROC AUC of 0.604 (95% CI 0.503–0.705) for predicting death at 56 days post-discharge. The augmented model showed an improved ROC AUC of 0.748 (95% CI 0.665–0.831; p = 0.009 for comparison between the two ROC AUCs) (Fig. 2b). Study hospital was not significantly associated with the primary outcome.

### Secondary outcome

The WHO multivariate model showed that being unable to walk unaided was the only danger sign that was independently associated with the secondary outcome (any one of the following: hospital admission for > 7 days, death in hospital, transfer to a tertiary level hospital, or transfer to a tuberculosis hospital) (Table 4). The augmented multivariate model showed that the secondary outcome was independently predicted by a lower CD4 count, a lower BMI, and being unable to walk unaided (Table 4). The WHO multivariate model showed a ROC AUC of 0.686 (95% CI 0.639–0.734) for predicting the secondary outcome. The augmented multivariate model showed an improved ROC AUC of 0.718 (95% CI 0.673–0.764; p = 0.001 for comparison between the two ROC AUCs) (Additional file 1: Figure S1A).

**Table 4:** WHO multivariate model (variables were WHO danger signs) and augmented multivariate model (variables were the WHO danger signs, and the following: CD4 count, body mass index, being on ART, hypotension, and confusion) for predicting the secondary outcome (any one of: hospital admission for > 7 days, death in hospital, transfer to a tertiary level hospital, or transfer to a tuberculosis hospital)

Variable	WHO multivariate model			Augmented multivariate model		
Category	aOR^a^	95% confidence interval	Wald’s p value	aOR	95% confidence interval	Wald’s p value
Respiratory rate > 30						
No	Referent group			Referent group		
Yes	1.19	0.79 to 1.79	0.408	1.21	0.79–1.85	0.376
Heart rate > 120						
No	Referent group			Referent group		
Yes	0.96	0.60 to 1.55	0.883	0.93	0.57–1.52	0.722
Temperature > 39 °C						
No	Referent group			Referent group		
Yes	1.01	0.60 to 1.68	0.980	0.97	0.57–1.64	0.899
Unable to walk						
No	Referent group			Referent group		
Yes	4.48	3.05 to 6.57	< 0.001	3.96	2.66–5.90	< 0.001
CD4 cells/µL^b^				0.85	0.73–0.98	0.025
BMI (kg/m^2^)^c^				0.97	0.93–1.00	0.073
On ART at admission						
No				Referent group		
Yes				1.28	0.84–1.96	0.249
Hypotensive^d^						
No				Referent group		
Yes				0.95	0.60–1.52	0.838
Confused						
No				Referent group		
Yes				1.48	0.87–2.53	0.146

Imputation set used due to missing CD4 and BMI data

^a^Adjusted odds ratio

^b^Increase in increments of 100 cells/µl

^c^Increase per 1 kg/m^2^

^d^Systolic blood pressure < 90 mmHg or diastolic blood pressure ≤ 60 mmHg

A sub-analysis of the WHO multivariate model among culture-positive tuberculosis participants, showed that being unable to walk unaided was the only WHO danger sign that independently predicted the secondary outcome (Additional file 1: Table S2). The augmented multivariate model showed that the secondary outcome was independently predicted by a lower CD4 count, a lower BMI, and being unable to walk unaided (Additional file 1: Table S2). The WHO multivariate model showed a ROC AUC of 0.697 (95% CI 0.631–0.763) for predicting the secondary outcome. The augmented multivariate model showed an improved ROC AUC of 0.750 (95% CI 0.68–0.813; p = 0.017 for comparison between the two ROC AUCs) (Additional file 1: Figure S1B). Study hospital was not significantly associated with the secondary outcome.

## Discussion

We found that being unable to walk unaided was the only WHO danger sign that predicted poor outcomes in a cohort of seriously ill HIV-infected participants hospitalised with cough. We established that several of the potential predictors of poor outcomes we selected a priori (lower BMI, lower CD4 count, and being on ART) improved the ability to predict poor outcomes. The high prevalence of tuberculosis in our cohort enabled us to also assess prognostic predictors in participants with culture-positive tuberculosis, with similar findings. To our knowledge ours is the first study to have evaluated the prognostic ability of the WHO danger signs, as well as other potential predictors, in the population of interest and in the sub-group with a final diagnosis of tuberculosis. In 2016 WHO updated the seriously ill algorithm to include the Xpert MTB/RIF assay and cough of any duration, [16] which makes our findings applicable to the updated algorithm, but cough is no longer a requirement.

The strong associations we found between being unable to walk unaided and the primary and secondary outcomes in our cohort indicate that this WHO danger sign is a simple, objective prognostic marker. The association between poor outcomes and both low CD4 counts and BMI was expected, as these are well-established prognostic markers in HIV infection [11, 17–20]. However, we expected that tachypnoea, confusion, and hypotension would be significant predictors of poor prognosis, given that these have been shown to be of prognostic value in patients with a variety of HIV-associated pulmonary infections [10, 11, 18, 21, 22].

Among the culture-positive tuberculosis participants, being unable to walk unaided, low BMI, low CD4 count, and being on ART remained strong prognostic indicators of poor outcomes. Most studies that assessed predictors of mortality among HIV-infected tuberculosis participants have been retrospective observational cohorts and evaluated outcomes at 6 months. We could not find any studies of inpatients that assessed predictors of 56-day mortality or poor outcome. Some of the independent predictors of poor outcomes in patients with HIV-associated tuberculosis from other studies include: age, [20, 23] sex, [20] ambulatory status, [24] WHO clinical stage 4, [23] BMI, [19] lower CD4 count, [20, 23, 24, 19, 25] ART, [20, 24, 25] cotrimoxazole prophylactic therapy, [24] haemoglobin, [19] and white cell count [19, 25].

Being on ART independently predicted death at 56 days post-discharge in the whole cohort, as well as among culture positive tuberculosis participants, which was an unexpected finding. Two studies reported a similar result to our findings, with increased mortality among patients who started ART within 3 months [26] and 6 months, [27] respectively, prior to their tuberculosis diagnosis. The increased mortality seen in patients started on ART a few months prior to the diagnosis of tuberculosis, as was the case in 8/27 of our participants on ART who died within 56 days post-discharge, could be due to the development of unmasking immune reconstitution inflammatory syndrome, which has been associated with an increased mortality [28]. Nine of nineteen participants who died within 56 days post-discharge and who were on ART for > 3 months had virologic failure, which is associated with an increased mortality [29]. Finally, patients with virologic failure typically have poor adherence, and their increased mortality in our study could in part be related to poor health-seeking behaviour and delayed presentation.

Our study has several limitations. First, we were unable to determine the cause of death in our participants. Second, all our participants had one or more WHO danger signs, and the lack of a control group without danger signs limited our ability to determine the prognostic value of the danger signs. Third, we did not have definitive reference standards to diagnose bacterial pneumonia and PJP. Finally, our cohort was from a setting with high prevalence of both HIV and tuberculosis. Our findings may not be generalizable to areas with lower incidence of tuberculosis or to children.

In conclusion, we assessed prognostic indicators of respiratory illness in our prospective cohort of HIV-infected participants presenting with a cough of any duration and WHO danger signs. We established that being unable to walk unaided, low BMI, and low CD4 count were strong predictors of morbidity and mortality. These findings were similar among culture-positive tuberculosis patients. The increased mortality we observed in participants on ART is an important determinant to keep in mind when assessing acute respiratory illness in HIV-infected patients. Future research should focus on filling these gaps and validating our results in different study populations.

## Additional file

**Additional file 1.** Additional figures and tables.
