# Supplementary material for: Prognostic indicators in the World Health Organization’s algorithm for seriously ill HIV-infected inpatients with suspected tuberculosis
Source: AIDS Res Ther. 2018 Feb 12;15:5. doi: 10.1186/s12981-018-0192-0 (PMC5808414; doi:10.1186/s12981-018-0192-0)
Supplement: Supplementary file 1 — Additional file 1. Additional figures and tables. [file 12981_2018_192_MOESM1_ESM.docx]

# Supplementary Appendix:

# **Prognostic indicators in the world health organization’s algorithm for seriously ill hiv-infected inpatients with suspected tuberculosis**

Rulan Griesel^1^, Annemie Stewart^1^, Helen van der Plas^2^, Welile Sikhondze^2^, Marc Mendelson^2*^, Gary Maartens^1*^

^1^Division of Clinical Pharmacology, Department of Medicine, University of Cape Town, South Africa

^2^Division of Infectious Diseases and HIV Medicine, Department of Medicine, University of Cape Town, South Africa

*These two authors contributed equally

**Supplementary Figure 1** Comparison of area under the curve (AUC) for the receiver operating characteristic (ROC) for the WHO multivariate model (variables were WHO danger signs) and the augmented multivariate model (variables were the WHO danger signs, and the following: CD4 count, body mass index, being on ART, hypotension, and confusion) for predicting a secondary outcome (any one of: hospital admission for >7 days, died in hospital, transfer to a tertiary level hospital, or transfer to a tuberculosis hospital) in all participants (A) and in participants with culture-positive tuberculosis (B).


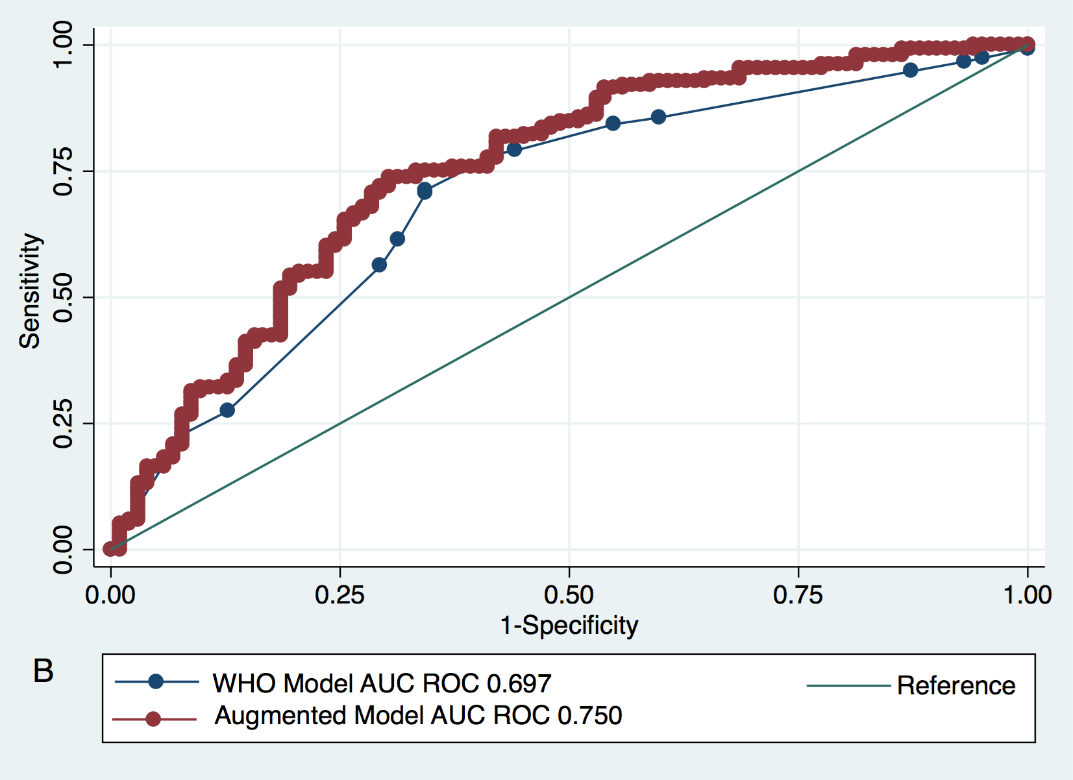

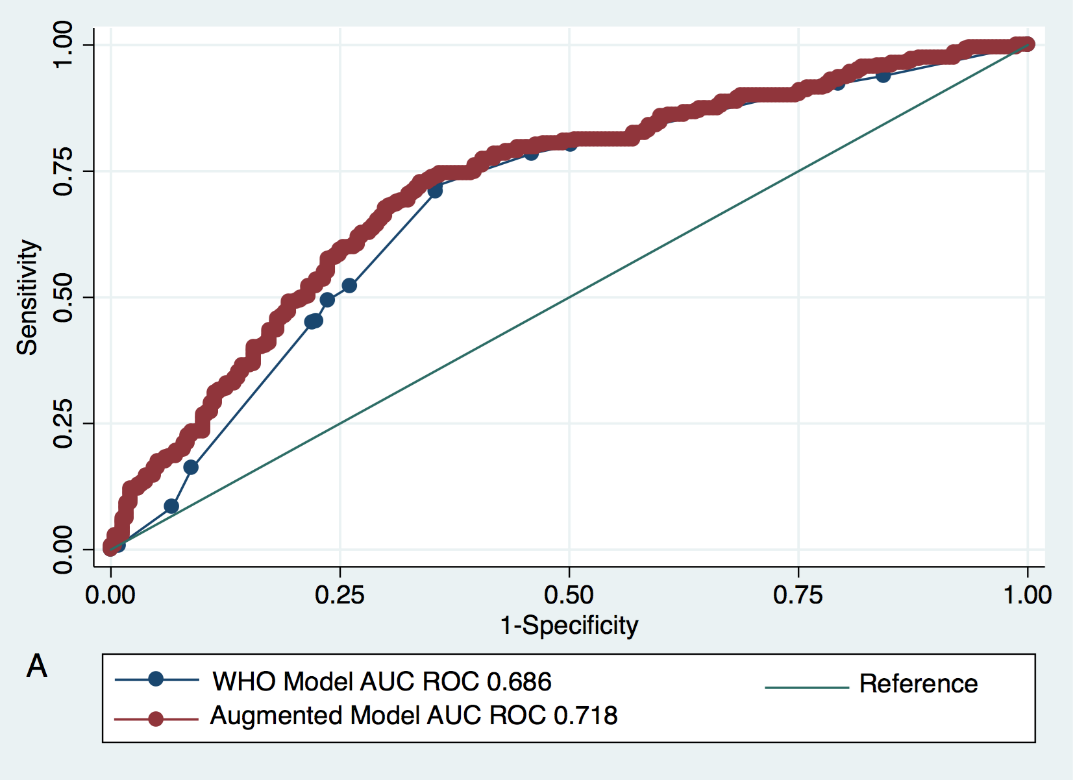


**Supplementary Table 1** WHO multivariate model (variables were WHO danger signs) and augmented multivariate model (variables were the WHO danger signs, and the following: CD4 count, body mass index, being on ART, hypotension, and confusion) for predicting death at 56 days, in participants with culture-positive tuberculosis. Imputation set used due to missing CD4 and BMI data.

| **Variable** | | | **WHO Multivariate Model** | | | **Augmented Multivariate Model** | | | |
| --- | --- | --- | --- | --- | --- | --- | --- | --- | --- |
|  | Category | | aOR^a^ | 95% confidence interval | Wald’s p-value | aOR^a^ | 95% confidence interval | | Wald’s p-value |
| **Respiratory Rate >30** | | | | |  |  | |  |  |
|  | | No | *Referent group* | |  | *Referent group* | | |  |
|  | | Yes | 0.88 | 0.42 to 1.85 | 0.730 | 0.79 | | 0.36 to 1.75 | 0.564 |
| **Heart Rate >120** | | | | |  |  | |  |  |
|  | | No | *Referent group* | |  | *Referent group* | | |  |
|  | | Yes | 1.07 | 0.41 to 2.79 | 0.894 | 0.84 | | 0.30 to 2.34 | 0.740 |
| **Temperature >39°C** | | | | |  |  | |  |  |
|  | | No | *Referent group* | |  | *Referent group* | | |  |
|  | | Yes | 0.81 | 0.31 to 2.09 | 0.662 | 0.62 | | 0.23 to 1.67 | 0.341 |
| **Unable to walk** | | | |  |  |  | |  |  |
|  | | No | *Referent group* | |  | *Referent group* | | |  |
|  | | Yes | 2.23 | 0.93 to 5.39 | 0.073 | 1.82 | | 0.71 to 4.65 | 0.211 |
| **CD4 cells/µL^b^** | | |  |  |  | 0.34 | 1.18 to 0.66 | | 0.001 |
| **BMI (kg/m^2^)^c^** | | |  |  |  | 0.97 | 0.88 to 1.07 | | 0.512 |
| **On ART at admission** | | | | |  |  |  | |  |
|  | | No |  | |  | *Referent group* | | |  |
|  | | Yes |  |  |  | 2.35 | | 1.03 to 5.32 | 0.042 |
| **Hypotensive^d^** | | |  |  |  |  | |  |  |
|  | | No |  | |  | *Referent group* | | |  |
|  | | Yes |  |  |  | 1.87 | | 0.81 to 4.35 | 0.143 |
| **Confused** | | |  |  |  |  | |  |  |
|  | | No |  | |  | *Referent group* | | |  |
|  | | Yes |  |  |  | 1.23 | | 0.50 to 3.07 | 0.650 |

^a^ Adjusted odds ratio, ^b^ Increase in increments of 100 cells/µl, ^c^ Increase per 1kg/m^2^, ^d^ Systolic blood pressure <90 mmHg or diastolic blood pressure ≤60 mmHg

**Supplementary Table 2** WHO multivariate model (variables were WHO danger signs) and augmented multivariate model (variables were the WHO danger signs, and the following: CD4 count, body mass index, being on ART, hypotension, and confusion) for predicting a secondary outcome (any one of: hospital admission for >7 days, death in hospital, transfer to a tertiary level hospital, or transfer to a tuberculosis hospital) in participants with culture-positive tuberculosis. Imputation set used due to missing CD4 and BMI data.

| **Variable** | | | **WHO Multivariate Model** | | | **Augmented Multivariate Model** | | | |
| --- | --- | --- | --- | --- | --- | --- | --- | --- | --- |
|  | Category | | aOR^a^ | 95% confidence interval | Wald’s p-value | aOR^a^ | 95% confidence interval | | Wald’s p-value |
| **Respiratory Rate >30** | | | | |  |  | |  |  |
|  | | No | *Referent group* | |  | *Referent group* | | |  |
|  | | Yes | 0.79 | 0.45 to 1.38 | 0.405 | 0.71 | | 0.39 to 1.29 | 0.262 |
| **Heart Rate >120** | | | | |  |  | |  |  |
|  | | No | *Referent group* | |  | *Referent group* | | |  |
|  | | Yes | 1.31 | 0.65 to 2.65 | 0.450 | 1.26 | | 0.60 to 2.64 | 0.534 |
| **Temperature >39°C** | | | | |  |  | |  |  |
|  | | No | *Referent group* | |  | *Referent group* | | |  |
|  | | Yes | 0.86 | 0.45 to 1.66 | 0.653 | 0.71 | | 0.36 to 1.42 | 0.336 |
| **Unable to walk** | | | |  |  |  | |  |  |
|  | | No | *Referent group* | |  | *Referent group* | | |  |
|  | | Yes | 4.71 | 2.70 to 8.21 | <0.001 | 4.19 | | 2.34 to 7.51 | <0.001 |
| **CD4 cells/µL^b^** | | |  |  |  | 0.65 | 0.49 to 0.87 | | 0.003 |
| **BMI (kg/m^2^)^c^** | | |  |  |  | 0.93 | 0.87 to 0.99 | | 0.017 |
| **On ART at admission** | | | | |  |  |  | |  |
|  | | No |  | |  | *Referent group* | | |  |
|  | | Yes |  |  |  | 1.41 | | 0.75 to 2.65 | 0.282 |
| **Hypotensive^d^** | | |  |  |  |  | |  |  |
|  | | No |  | |  | *Referent group* | | |  |
|  | | Yes |  |  |  | 0.84 | | 0.43 to 1.63 | 0.601 |
| **Confused** | | |  |  |  |  | |  |  |
|  | | No |  | |  | *Referent group* | | |  |
|  | | Yes |  |  |  | 0.69 | | 0.34 to 1.43 | 0.318 |

^a^ Adjusted odds ratio, ^b^ Increase in increments of 100 cells/µl, ^c^ Increase per 1kg/m^2^, ^c^ Systolic blood pressure <90 mmHg or diastolic blood pressure ≤60 mmHg
